# The Essentials in Teaching

**Published:** 1901-03

**Authors:** 


### THE ESSENTIALS IN TEACHING.

IN another part of this number will be found an interesting and timely article on "Original Investigation not a Necessary Quality of the Teacher."

This statement and the arguments brought to bear to enforce them will necessarily arouse a wide diversity of opinion. The question, however, is one of serious importance to all teaching bodies, the students directly interested, and indirectly also to the general public.

Without attempting to follow the line of thought adopted by our much-esteemed correspondent, the writer will consider the question from the practical stand-point of experience.

Teaching is recognized to be the power to draw out the best there is in the pupil; in other words, to educate to a position approximating the standard held by the teacher. To accomplish this properly means a quality of mind not generally possessed,—that of descending to the plane of intellect of the student and, for the time being, occupying his position, thinking his thoughts, and combating the difficulties presenting to his mind. Then, having thus, as it were, transformed himself from the superior to the inferior, he will naturally voice his instruction in a language comprehensible to the receiving mind. This would seem to be the foundation upon which to build for future service, and this once established, other things can be added.

The next most important qualification is that the teacher should be a thorough master of his subject. It does not follow that this will qualify a person to teach, but success can never result from imperfect knowledge.

While it is not absolutely essential that a teacher should be an original investigator upon professional topics, he will proportionately fall far below a high standard if he depends entirely upon the work of others. It is equally true that the investigator, the laboratory worker, who depends upon that alone, may fail, and generally does fail, in his efforts to impart knowledge. If the teacher be a lecturer, he must have so mastered his subject that he will cease to depend upon his notes or his manuscript.

Considering seriatim the points named, the writer would first hold the mastery of the subject to be taught as the prime essential qualification of the teacher, but it is a well-known fact that many have been chosen for positions in professional and other schools largely upon the fact that they have proved themselves masters in special branches, and it was taken for granted that this profound knowledge or manipulative skill not only justified the selection, but insured thorough teaching. The examples, abounding everywhere, of failures in this class prove quite conclusively that pedagogical excellence is not dependent upon the exact knowledge or skill acquired by the individual.

There is, probably, no problem more difficult of solution than the selection of proper teachers. It is an ever-present question in all schools, colleges, and universities, and is more and more giving managers of dental colleges grave anxiety. The present curricula demand an altogether different class of teachers from those of the past. Are these being developed? Men are not difficult to find who are skilled in the several practical branches; indeed, in some instances they may be superior to the average expert, but are utterly incapable of imparting the knowledge and skill they possess. So exceedingly rare is it to find combined skill and power to impart, that dental colleges, as well as all institutions of learning, are searching the country over to find those possessing the qualities demanded. Failure to find this class of mind forces a dependence upon an inferior article, to the injury of the product.

The time has not arrived in any dental school when pedagogical instruction can be added to the curriculum of the senior year, but some training of how to teach is especially desirable at the present time. The young men go out into the world with very crude ideas in this direction, and, possibly, in a very short time are filling professional positions with very little conception either of the responsibility or of what is required of one occupying the position of a teacher.

The question largely dwelt upon by our correspondent, whether original investigation helps or hinders the teacher, is one not easily answered. Some minds are capable of so thoroughly absorbing the facts obtained by others and making them their own, that they seem able to readily transfer this knowledge to others. Memory plays here an important part. The man who can read a book and store the facts therein contained away for future reference has a power possessed but by the few, and as he gives out this second-hand knowledge he will, in all probability, astound his hearers with his profundity. Is this teaching? To the writer it corresponds to the auction sale of long-used furniture, the life and beauty has measurably departed.

When ability to experiment in the laboratory is combined with a faculty of analysis to deduce results from the facts obtained, and to be able from time to time to repeat the investigations of others, and at the same time, in simple language, to be able to impart this knowledge, it gives to the instruction a life and power not to be obtained by mere memorizing of facts from books. So impressed has the writer been with the truth of this in his own experience, that he invariably feels a deficiency where it has been impossible for him to duplicate statements made upon laboratory investigation. There is that unexplainable something underlying the teaching that leads him to feel that the only true foundation for all teaching is to first do the work and then to endeavor to discover the best way to impart the knowledge obtained. It is recognized that in many instances this is impossible, and the teacher will be obliged to render a doubtful service to that extent; but while this is true, it does not follow that original investigation is not a necessary quality of the teacher.

The societies that have sprung up in our colleges of recent years have done an excellent work in training the members to an increased ability in extemporary speaking. This is one of the essentials of good teaching, and every encouragement should be extended for further progress in this direction. The dental education of the future will depend on these young men, and if they are able to impart the knowledge possessed without extended resort to written lectures, it will be one step, and an important one, gained in ability to instruct. There is no method quite equal to reading from a manuscript to destroy all true teaching. The monotony of a poorly read lecture becomes unbearable, and the tired brain of the student refuses to absorb the matter presented. Cooking undoubtedly spoils much of our physical food, and the method of preparation of many lectures results in mental indigestion to the hearers. The excuse will be made that all are not capable of extemporary discourses. This is probably true of nearly all novices in teaching, but it is not difficult to combine the two, written and extemporary teaching. It is a great relief to the student to have the teacher leave his manuscript and speak for a few minutes, by way of explanation, on some important topic. He will notice at once the sudden stirring of the class from apathy to interest.

The importance of this whole question felt by all teachers in dentistry is not confined to their work. It is one that possesses paramount importance in the minds of all classes of instructors, and the same difficulties are met with elsewhere as are found in professional work. Means are furnished for pedagogical instruction in the schools, but these are not yet attainable by professional teachers, and we will doubtless go on in the old crude way for an indefinite period, taking the man and placing him at the head simply because he is a good operator, a generally good prosthetic worker, or a specialist in one of the several branches of medical practice. When an individual can prove that he can make of the raw student material, a good operator, a good mechanician, a good anatomist, he has proved his fitness for the position of teacher in these several branches.

The true teacher, therefore, in the opinion of the writer, combines not memory alone, or power to marshal in order the work of others, but mingling this with analytical reasoning and ability to seek truth from original sources, he will be able to lucidly clear up the most abstruse problems to the slowest mind in his class. This should be the ideal attainment of all who aim to impart the accumulated wisdom belonging to their profession.

The foregoing is confined mainly to didactic teaching, but applies with equal force to direct individual teaching. The didactic method has been tried for generations in all professional schools, and has been found so defective that in medicine and dentistry individual teaching is more and more taking its place. The old, doubtless, will be continued to elucidate principles, but beyond that it has lost vitality as a factor in presenting the practical side of professional work.

